# Reimbursement of licensed cell and gene therapies across the major European healthcare markets

**DOI:** 10.3402/jmahp.v3.29321

**Published:** 2015-09-30

**Authors:** Jesper Jørgensen, Panos Kefalas

**Affiliations:** Cell Therapy Catapult Limited, Guys Hospital, London, UK

**Keywords:** Pricing and reimbursement, market access, healthcare funding, advanced therapy medicinal products (ATMPs), specialised care, health technology assessment (HTA), cross country comparison, Big5EU (France, Germany, Italy, Spain and the United Kingdom)

## Abstract

**Objective:**

The aim of this research is to identify the pricing, reimbursement, and market access (P&R&MA) considerations most relevant to advanced therapy medicinal products (ATMPs) in the Big5EU, and to inform their manufacturers about the key drivers for securing adoption at a commercially viable reimbursed price.

**Methodology:**

The research was structured following three main steps: 1) Identifying the market access pathways relevant to ATMPs through secondary research; 2) Validating the secondary research findings and addressing any data gaps in primary research, by qualitative interviews with national, regional, and local-level payers and their clinical and economic advisors; 3) Collating of primary and secondary findings to compare results across countries.

**Results:**

The incremental clinical benefit forms the basis for all P&R&MA processes. Budget impact is a key consideration, regardless of geography. Cost-effectiveness analyses are increasingly applied; however, only the United Kingdom has a defined threshold that links the cost per quality-adjusted life year (QALY) specifically and methodologically to the reimbursed price. Funding mechanisms to enable adoption of new and more expensive therapies exist in all countries, albeit to varying extents. Willingness to pay is typically higher in smaller patient populations, especially in populations with high disease burden. Outcomes modelling and risk-sharing agreements (RSAs) provide strategies to address the data gap and uncertainties often associated with trials in niche populations.

**Conclusions:**

The high cost of ATMPs, coupled with the uncertainty at launch around their long-term claims, present challenges for their adoption at a commercially viable reimbursed price. Targeting populations of high disease burden and unmet needs may be advantageous, as the potential for improvement in clinical benefit is greater, as well as the potential for capitalising on healthcare cost offsets. Also, targeting small populations can also help reduce both payers’ budget impact concerns and the risk of reimbursement restrictions being imposed.

This article describes the variation in pricing, reimbursement, and market access (P&R&MA) processes for cell and gene therapies across and within the Big5EU (France, Germany, Italy, Spain, and the United Kingdom). Besides geography, we also describe the P&R&MA impact of the magnitude of the incremental benefit of the novel therapy compared with existing therapeutic approaches, the size of the target patient population, as well as the availability of additional funding for new and costly therapies. We concentrate on those cell and gene therapies that are licensed by the European Medicines Agency as advanced therapy medicinal products (ATMPs), which are intended for use within the hospital setting (inpatient and outpatient) and reimbursed by public healthcare systems.

Across the Big5EU, licensed ATMPs go through the same processes for P&R&MA and funding as ‘conventional’ pharmaceuticals. Over time, the assessment of reimbursed price for innovative licensed therapies has shifted towards value-based models; cost-plus pricing approaches are giving way to competitor-based approaches (e.g., reference-pricing groups operating in multiple European countries), which are now largely applied to undifferentiated or poorly differentiated products (see Table 1).

**Table 1:** Methodologies for pharmaceutical pricing

	Cost based		Competitor based		Value based	
What is it?	•	Price based on costs, expected sales, and margins	•	Price driven by competition	•	Price based on comparative effectiveness
Examples	•	Cost-plus pricing	•	Penetration pricing	•	Price based on cost–utility
			•	Reference group pricing		
Comments	•	Becoming obsolete	•	Enforced for undifferentiated products	•	Typical for differentiated products
	•	Exception: unlicensed ATMPs				

Value-based assessments explore the added value of a novel therapy compared with existing therapeutic alternatives (i.e., standard of care [SOC] or best supportive care). By quantifying and monetising the magnitude of the added value, the therapy's reimbursed price potential is determined (1). Thus, value-based assessments provide a link between therapy benefits (for the patient and the healthcare system) and the willingness to pay and adopt.

The methods by which added value is captured and translated into a reimbursed price varies by geography. The most common lever employed across all Big5EU markets is the magnitude of the incremental clinical benefit; economic factors are then considered (e.g., cost-effectiveness [CE], budget impact) with willingness to pay often being influenced by size of target patient population and the perceived level of disease burden. Relevant domestic pricing benchmarks (where available) are accounted for either directly by informing the baseline price over which a price premium is added or indirectly through the cost of the displaced therapy in health economic models. International price referencing is leveraged to varying extents in all countries, except the United Kingdom (2). Additional factors such as contribution to GDP, lobbying, involvement of patient advocacy groups as well as ethical, equality, and equity considerations can also impact final P&R&MA outcome (see Fig. 1).

**Fig. 1:**
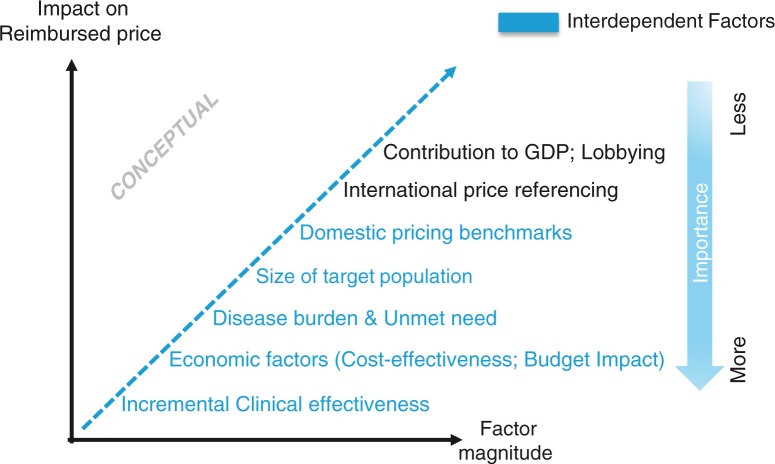
Factors impacting pharmaceutical price build up (conceptual).

Central to value-based assessments is the availability of comparative clinical data, and direct head-to-head comparisons are the gold standard for the purpose of health technology assessments (HTAs). However, indirect comparisons are increasingly used, especially in situations where patient recruitment and ethical considerations present challenges with the inclusion of comparator arms in clinical trials (3). Comparative effectiveness can also be strengthened through modelled data; however, the acceptability of such data varies across the Big5EU.

In cases where the clinical and economic outcomes associated with the SOC, is not well documented in the public domain, generation of comparative evidence may also be necessary to demonstrate incremental benefit of new treatments. This is particularly relevant for treatments targeting very rare diseases or niche subpopulations within larger therapy areas.

Despite their substantial therapeutic potential, ATMPs face specific P&R&MA challenges compared with other therapeutic categories:

1. ATMPs come with high manufacturing costs, which dictate a high target price in order to be commercially viable; even when CE is demonstrated, budget impact can be a concern.
2. The incremental benefit claims can extend over a longer horizon than is supported by clinical trial data at launch; biological plausibility suggests that a single or finite number of treatments can potentially provide life-long benefits. This is likely to be the case across a range of technology classes where cell replacement or long-term gene modification are targeted. One recent example is the case with the approved gene therapy ATMP Glybera (4)
3. Where novel interventional procedures are required to deliver an ATMP, these may need to undergo a separate HTA to that of the ATMP itself, sometimes as a prerequisite to the assessment of the actual ATMP (e.g., in England, an Interventional Procedure Guidance issued by National Institute for Health and Care Excellence [NICE] needs to precede a technology appraisal if the technology is being delivered to the body in a novel way) (5). ATMPs relying on intricate interventional procedures for their administration are likely to be restricted to centres of excellence only. Furthermore, the reimbursed price potential of an ATMP is impacted by the cost of the required interventional procedures.
4. Autologous ATMPs present additional challenges for hospital care configuration and financing as they have the potential to disrupt existing treatment algorithms by introducing additional steps (e.g., bone marrow aspiration); therefore, assessments of such therapies can demand additional considerations including reallocation of healthcare resources and re-engineering of existing service delivery processes.

Even if these barriers are overcome, and a positive recommendation for use is given by national and/or regional (where applicable) HTA bodies, healthcare providers may be slow to introduce and use new products and/or configure services to ensure adoption. For manufacturers, this creates additional challenges, beyond the evaluation process, both in terms of forecasting and for actual adoption of their products.

The aim of this research is to identify the pricing, reimbursement, and market access (P&R&MA) considerations most relevant to ATMPs in the Big5EU, and to inform their manufacturers about the key drivers for securing adoption at a commercially viable reimbursed price.

## Methodology

The research was structured following three main steps.

1. First, the market access pathways relevant to ATMPs were identified through secondary research, including the key stakeholders involved in national, regional, and local levels, and the decision analysis framework for P&R&MA. The secondary research included a review of the websites of the various national public health institutions. This was complemented by a research for peer-reviewed articles on MEDLINE and relevant recent ISPOR (International Society for Pharmacoeconomics and Outcomes Research) conferences. Additional Internet searches using Google and Google Scholar were also conducted (grey literature was also searched for upon the advice of experts).
2. These findings were validated in primary research in each of the Big5EU countries, by qualitative interviews with healthcare system experts in the field of market access of health products including national, regional, local-level payers and their clinical and economic advisors. Separate discussion guides were developed to ensure country-specific, yet standardised discussions. The results reported here cover country-specific P&R&MA processes, as well as funding for ATMPs, and views on possible future developments.
  - In the United Kingdom specifically, we also leveraged our participation in the Regenerative Medicine Expert Group to generate relevant insights. The remit of the Regenerative Medicine Expert Group has been to develop an NHS regenerative medicine strategy so that the NHS is fully prepared to adopt and deliver these innovative treatments.
3. Finally, the secondary and primary research findings were collated to provide an overview of the different routes to market in the respective Big5EU countries.

## Results

### Centralisation of decision-making

Across the Big5EU there is variation in the degree of centralisation of the P&R&MA processes. France and Germany are relatively centralised markets with decisions made at national level being largely implemented at regional and local level. This means that the P&R&MA process in these countries is less fragmented, as an approval on national level generally encourages formulary inclusion and funding at the hospital level. In France, the 26 regional health agencies all distribute funding to hospitals, but have otherwise a limited role on P&R&MA decision-making. In Germany the 132 health insurers, or *sickness funds* (Krankenkassen, KKs) (6), all distribute funding to hospitals, and have little say in terms of the level of funding, except for new and expensive hospital treatments that exceed the existing funding levels. In these cases, the KKs decide whether or not to provide funding under the NUB scheme, and at what levels.

On the contrary Italy and Spain are largely decentralised. A reimbursed ceiling price is negotiated at national level in both markets but ultimately the final pricing, funding, and adoption decision lies with each of the autonomous regions (21 in Italy and 17 in Spain) (7, 8). As part of the regional negotiations, reimbursed price level agreed at national level can be negotiated down, and occasionally some therapies may fail to be adopted and secure funding in certain regions. Exceptions apply, for example, in the case of therapies that are granted ‘innovative classification’ by the Italian Medicines Agency at national level, which must be made available in all the autonomous regions.

The United Kingdom in particular has a strong regional structure through the devolved administrations of England, Northern Ireland, Scotland, and Wales, which are informed by different regional HTA agencies (NICE,^1^ Scottish Medicines Consortium [SMC], All Wales Medicines Strategy Group [AWMSG]), and regional commissioning bodies deciding on therapy adoption. In England, ATMPs are commissioned through NHS specialised services, which makes the local-level clinical commissioning groups less relevant to their P&R&MA.

### Pricing, reimbursement and market access in the Big5EU

Across the Big5EU, licensed ATMPs go through the same processes for P&R&MA and funding as ‘conventional’ pharmaceuticals, whereby new market entrants are assessed according to the comparative clinical effectiveness of the novel therapy versus a relevant comparator. However, different levers are applied in the different markets when differentiating product value is translated into price (see Table 2).

**Table 2:** Levers applied in price-setting for new therapies for NHS adoption in the Big5EU

Levers	France	Germany	Italy	Spain	The United Kingdom
1st order	Comparative clinical effectiveness of the novel therapy versus a relevant comparator in the given market				
2nd order	*With substantial added benefit (ASMR I-III):* International price referencing (EU4) + Cost-effectiveness	*With added benefit:* Budget impact Efficiency Frontier International price referencing (EU15)	Budget impact + International price referencing (cost-effectiveness: minor lever)		Cost-effectiveness
	*With comparable or minor added benefit (ASMR IV-V):* Domestic comparator price Price-volume agreements	*With no added benefit:* Domestic comparator price			

In the following paragraphs, we will describe in more detail the country-specific P&R&MA pathways for new, licensed ATMPs in the Big5EU (France, Germany, Italy, Spain, and the United Kingdom).

#### France

**Fig. 2:**
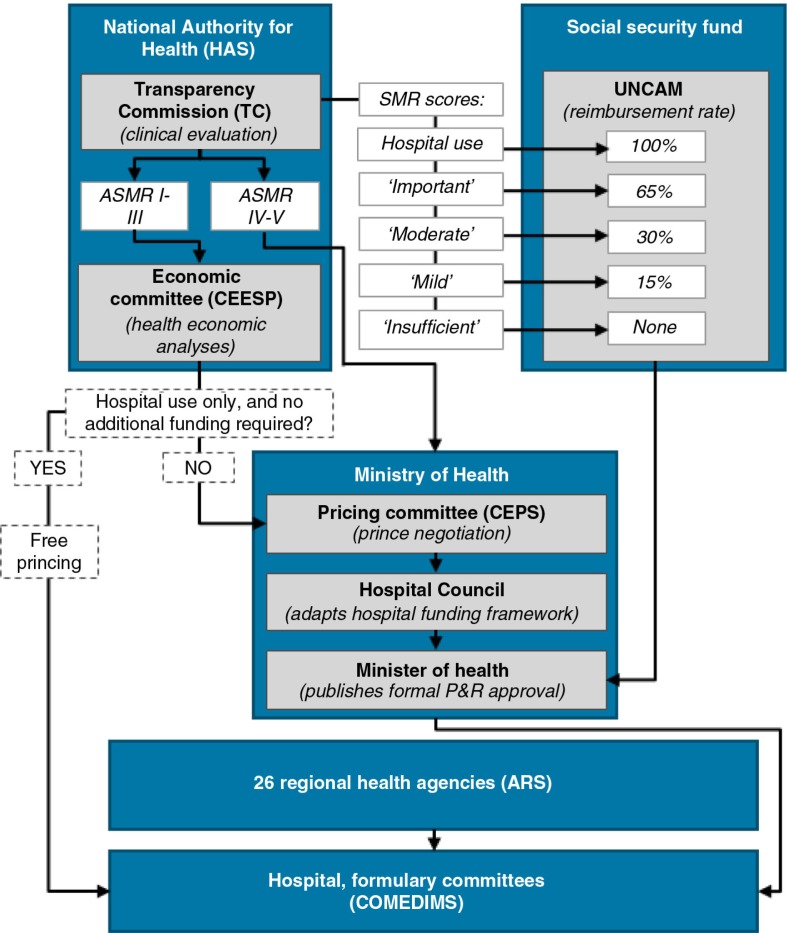
ATMP market access pathway in France.

The Transparency Commission (TC), a sub-division of the National Authority for Health (HAS), assesses clinical efficacy and safety, and concludes on the *actual benefit* (SMR), as well as the *improvement in actual benefit* (ASMR) versus an appropriate comparator. The SMR is used by the Social Security Fund's health insurance (UNCAM) to set reimbursement rate, whereas the ASMR is taken into account by the pricing committee (CEPS) under the Ministry of Health, when negotiating the reimbursed ceiling price (9). Therapies with both a substantial improvements in clinical benefit (ASMR I-III) *and* an estimated budget impact of >€20 million also undergo a CE evaluation by the economic commission (CEESP), which is used by CEPS in price negotiations (10). The Minister of Health publishes the final P&R decision based on TC and CEPS opinions. For ATMPs, which are mainly used in the hospital setting, the hospital formulary committees (COMEDIMS) also play a central role for market access, as they decide on formulary inclusion (see Fig. 2).

**Fig. 3:**
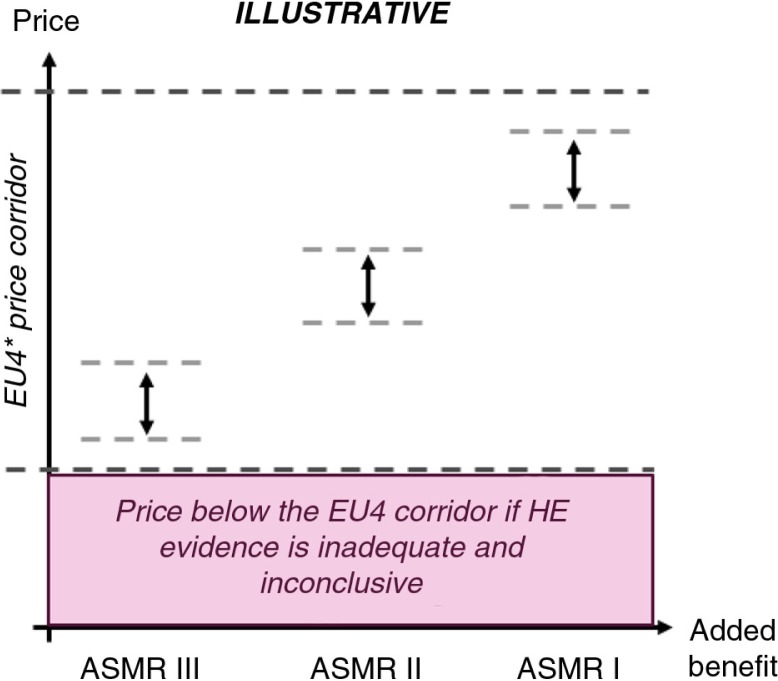
Pricing in France. *Germany, Italy, Spain, and the United Kingdom.

The CEPS negotiates price depending on the level of added benefit (ASMR) (11). Prices for therapies with only moderate improvements (ASMR IV-V) are negotiated based on domestic comparator prices, while prices for therapies with substantial improvements in clinical benefit (ASMR I-III) are benchmarked against the price of the same therapy in the EU4 (Germany, Italy, Spain and the United Kingdom). The ASMR score and CE analyses are used as levers to determine the acceptable price level for reimbursement within the EU4 price corridor (see Fig. 3). It should be emphasised that no willingness to pay threshold per quality-adjusted life year (QALY) gain has been defined in France, and the CEESP is not expected to be prescriptive in this respect.

Price/volume agreements are widely used to reduce uncertainty around budget impact. Price is commonly discounted stepwise at specified (confidential) volume thresholds, where greater discounts are applied for sales volumes beyond the defined thresholds. Rebates can also be applied, especially in the case of therapies, for which the CEESP evaluation does not present a strong CE case.

#### Germany

The Joint Federal Committee (G-BA) performs a clinical benefit assessment, commonly with input from the Institute for Quality and Efficiency in Healthcare (IQWiG), and this forms the starting point for P&R&MA negotiations for the majority of novel therapies. This so-called *early benefit assessment* 2 rates the incremental clinical effectiveness of the novel therapy versus the appropriate comparator (as defined by the G-BA), and subsequently, the National Association of Statutory Health Insurance Funds (GKV Spitzenverband) negotiates reimbursed price on behalf of the 132 sickness funds (health insurers) (12).

**Fig. 4:**
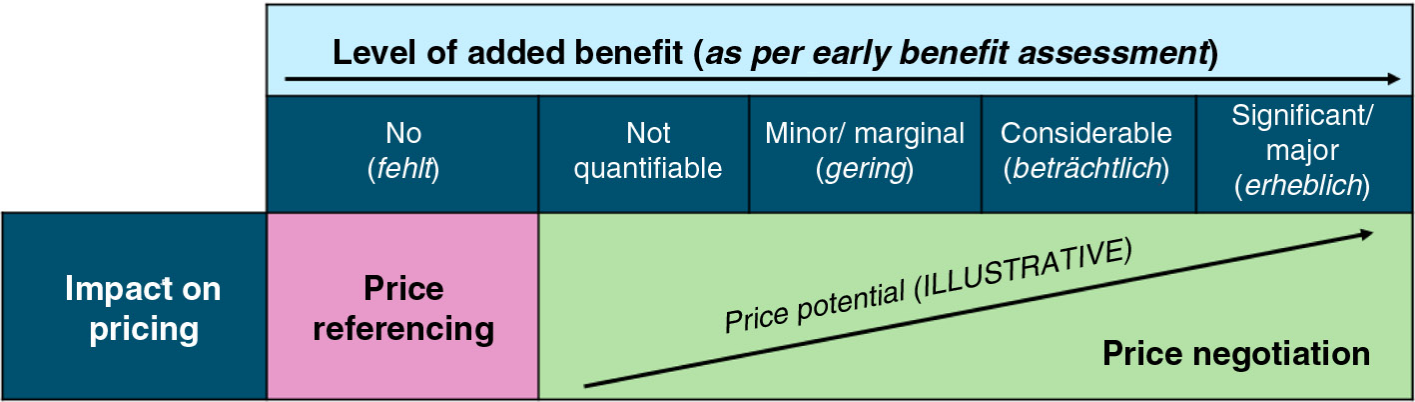
Early benefit assessment outcomes and impact on pricing.

New therapies enjoy free pricing for the first 12 months after launch (during which the early benefit assessment and price determination takes place), after which the reimbursed ceiling price is applied (12). Therapies with annual revenue <€1 M, or hospital-only therapies that are sufficiently covered by existing funding, also enjoy free pricing beyond the 12-month mark (13). In Germany, CE analyses (i.e., cost per QALY) play a limited role in P&R&MA; the results of the early benefit assessment and budget impact are the key drivers of the reimbursed price potential (see Fig. 4). International price referencing (based on a basket of 15 EU countries) can be applied by arbitration in cases where an additional benefit is recognised, but no agreement is reached in the price negotiations between the manufacturers and the GKV Spitzenverband (12).

**Fig. 5:**
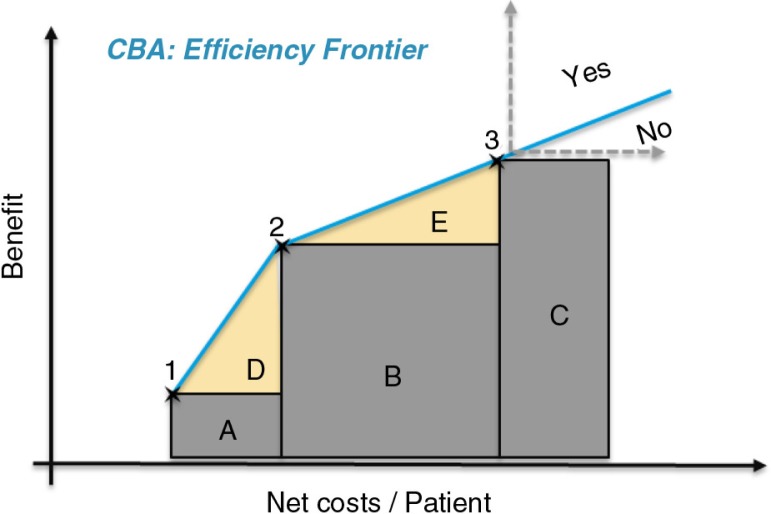
Cost–benefit assessment methodology (as explained by IQWIG) (14). The numbers 1-3 represent three existing therapies, and the blue line, the *efficiency frontier*, represents the willingness to pay, as illustrated by the adoption of more expensive, but more efficient therapies (i.e., from 1, via 2, to 3). New treatments that exceed the existing cost and benefit levels (beyond point 3) can be considered acceptable if the increase in net benefit and net costs are above the extension of the *benefit frontier*, that is, the extension of the willingness to pay from point 2 to 3, and beyond.

The main health economic analysis applied in Germany is budget impact; however, cost-benefit assessments (CBAs) are possible in two scenarios: 1) where the early benefit assessment concludes ‘no additional benefit’, but the product cannot be included in a reference price group (e.g., new mechanism of action); *or* 2) as a last resort, if the manufacturer rejects the EU15-based arbitration price (12). The principles of the CBA methodology as a tool to support decision-making is described in Fig. 5.

#### Italy

Italy is a highly decentralised country, where the 21 regions have substantial autonomy in terms of pricing, funding, and therapy adoption (7). P&R&MA is mainly negotiated according to the level of additional benefits, but in a non-prescriptive manner, which leaves great discretion for decision-makers at all levels to negotiate. The National Medicines Agency (AIFA) is the main decision-making body at the national level, which approves drug licenses, evaluates drugs for inclusion in the national formulary, and negotiates national ceiling prices (15). AIFA's Scientific Commission (CTS) evaluates the clinical value of new drugs and defines the reimbursed areas of use (hospital only, restrictions to subpopulations, etc.), whereas the pricing committee (CPR) negotiates prices and reimbursement conditions of new drugs based on CTS's opinion (16). AIFA also determines whether a new therapy is classified as ‘innovative’, based on disease severity, availability of treatment options, and level of clinical efficacy. Being classified as ‘innovative’ is of major strategic importance, as all ‘innovative’ products must be included in all the regional formularies across Italy. Budget impact is a key consideration, and price negotiations for high-cost therapies can be delayed tactically by AIFA to minimise the financial exposure to the NHS. Further, across-the-board price cuts, budget caps, and mandatory paybacks are also common.

Real-world evidence collected through registries is often a requirement for the market access of innovative therapies in Italy (especially in the oncology area) (17); P&R&MA conditions can then be revised depending on the outcomes generated.

Risk-sharing agreements (RSAs) between manufacturers and the Italian NHS are used extensively in specialised care, and are often coupled with requirements for real-world evidence generation. RSAs (also called innovative P&R agreements) can help mitigate payer uncertainty where there is a lack of long-term data at launch. Under RSAs, funding and use is commonly restricted to certain centres, and real-world patient outcomes must be recorded in product-specific AIFA registries (17). Additional discounts and/or rebates – typically maintained confidential – may apply on top of mandatory statutory discounts and can be linked to reaching certain milestones, for example, treatment response (*payment for performance*), as captured by the product registries.

#### Spain

**Fig. 6:**
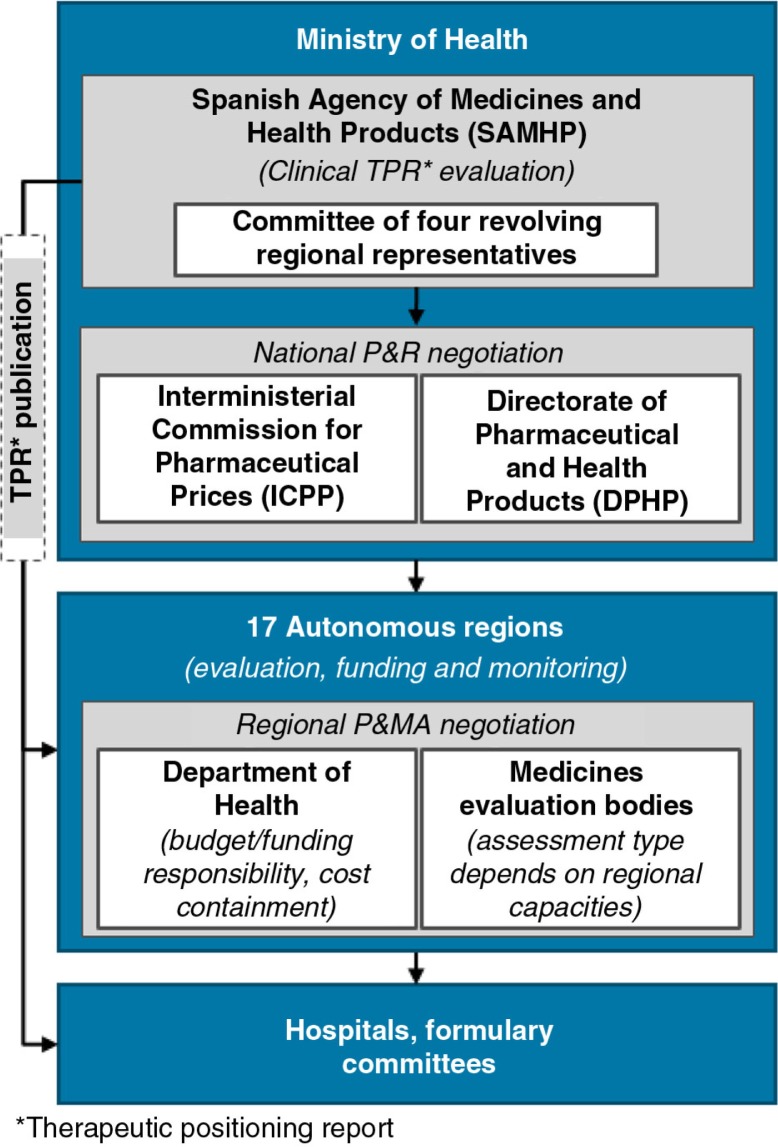
Market access pathway in Spain.

As in Italy, P&R&MA in Spain is highly decentralised with regional health authorities playing a leading role in healthcare provision and funding (8). At the national level, the Spanish Agency of Medicines and Health Products (SAMHP) is the competent regulatory authority and also develops the Therapeutic Positioning Report (TPR), comprising an evaluation of additional clinical benefit and definition of target population. Four regions are represented (on a revolving basis) in SAMHP for the development of the TPR (18). The TPR publication is key for market access, as it defines the areas of reimbursed use; however, the TPR is not binding; the 17 regions make binding decisions on funding and provision of care, and on what treatments to include on the regional formularies (see Fig. 6).

The Inter-ministerial Committee of Pharmaceutical Prices (ICPP) negotiates the national reimbursed ceiling price with the manufacturer (19) based on three documents: 1) the TPR as described above; 2) the price application submitted by the manufacturer, outlining proposed price, sales forecasts, R&D costs of the treatment; and 3) the value dossier submitted by the manufacturer, detailing clinical and health economic value proposition, budget impact, CE, and GDP contribution (20–22). Pricing authorities may use domestic comparator drugs as pricing benchmarks where relevant and/or the reimbursed price of the novel therapy in other EU countries (22). Furthermore, the clinical comparator and the price comparator may not necessarily be the same; the cheapest available treatment option in the therapy area is the starting point for price negotiations, even in cases where this may not be the clinical comparator. Also, pricing authorities have been known to reference the lowest available prices of the new therapy in the Euro zone during negotiations. The regional authorities then engage in the second tier price negotiations, where the national ceiling price typically is negotiated down.

The fact that four of the Spanish regions are represented in the TPR development on a revolving basis opens up the use of different assessment methodologies and value-driving criteria depending on which regions are represented at the time of launch.

Although the submission of a CE analysis by the manufacturer is compulsory for the national assessment, its P&R impact is limited; strict budget constraints dictate a highly cost-sensitive pricing environment, where budget impact is the key driver of negotiations at all levels. Also, a lack of clearly defined decision-making criteria leaves substantial room for negotiations and presents a risk for market access delays.

Regional health ministries make binding P&MA decisions for their populations; however, their assessment methodologies and capabilities vary greatly (8). Catalonia, the Basque Country, Madrid, and Andalusia perform the most advanced assessments, and commonly re-evaluate therapies for funding, P&MA (after the national assessment), which can cause market access delays.

#### UK

**Fig. 7:**
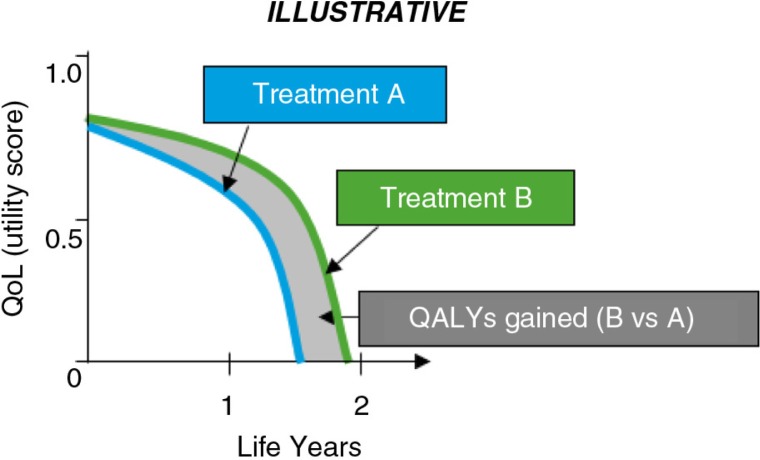
Comparison of quality-adjusted life years (QALYs) across two hypothetical treatments (A and B).

In England, the Department of Health (DoH) makes the final decision on P&R, based on assessments performed by the NICE. NICE applies a value-based assessment leveraging clinical effectiveness and CE considerations when developing recommendations for NHS adoption. Whereas NICE undertakes a variety of assessments, only two types result in binding obligations for NHS commissioning: 1) the Technology Appraisals (TA) and 2) the highly specialised technology evaluations (HSTE). The assessment methodology applied in NICE TAs is that of cost utility, that is, a CE analysis where effectiveness is measured in terms of QALYs, regardless of therapy area. QALYs account not only for the life years lived, but also the life-quality (utility) experienced by the patient (see Fig. 7).

By comparing the incremental costs of introducing a new treatment to the incremental benefits (QALYs) it delivers over the SOC, an incremental cost-effectiveness ratio (ICER) is calculated. ICER values below £30,000^3^ generally are considered cost-effective and associated with favourable recommendations for NHS adoption (23).

$$\text{ICER}=\frac{\left(\text{Cost of treatment B}-\text{Cost of treatment A}\right)}{\left(\text{QALY of treatment B}-\text{QALY of treatment A}\right)}$$

A higher threshold of up to £50,000/ QALY can be considered for end-of-life treatments with small populations (24), provided that an increase in survival by at least 3 months over the SOC is demonstrated. Very rare conditions with high disease burden tend not to be selected for the NICE TA programme but are more suitable for NICE HSTE (25); exceeding the ICER threshold does not necessarily impact the HSTE recommendation.

For ATMPs that do not undergo NICE TA or HSTE, the commissioning decision in England lies entirely with NHS England and more specifically with the NHS specialised services, with input from the Clinical Reference Groups; for therapies targeting rare diseases, the Rare Diseases Advisory Group is also consulted (5). Under the NHS specialised services assessment framework, meeting the ICER threshold of £30,000 per QALY is not an explicit criterion for adoption.

Non-binding NICE guidance (e.g., Interventional Procedures Guidance, or Medical Technologies Evaluation) can help inform the decision-making of the NHS commissioners, but without the obligation to be implemented.

In principle, free pricing applies throughout the United Kingdom; however, it is curbed by limits set on profitability by return on capital and return on sales (for entire company portfolio rather than individual products) (26). However, this freely set price (often quoted as ‘official list price’) is not guaranteed to be reimbursed, especially in the case of innovative therapies for which the willingness to pay and adopt is influenced by CE considerations. Therefore in the United Kingdom, it is important to differentiate between ‘official list price’ and ‘reimbursed price’ as it is the latter that the NHS pays the manufacturer. In cases where the therapy is found not to be cost-effective or there is insufficient evidence to conclude on CE, patient access schemes (PAS) can be pursued by the manufacturer, where a price revision is negotiated with DoH to improve the CE of the therapy (27). These PASs can either take the form of discounts or be outcome-based, for example, payment-for-performance RSAs. Unlike outcomes-based PAS, when the PAS is a mere discount, its terms are to be kept confidential. Such confidentiality can safeguard price potential (in favour of the manufacturer) in markets that use price in the United Kingdom for international price referencing. However, this safeguard depends on the stringency of such confidentiality, over which occasional concerns have been expressed.

Although commissioning decisions made by NHS England influence those made by NHS Northern Ireland, NHS Scotland and NHS Wales, these UK regions maintain autonomy with respect to P&R&MA processes. Regional HTA bodies such as the SMC and AWMSG do conduct separate assessments to those conducted by NICE and on many occasions they may decide differently on a given therapy's adoption.

### Availability of top-up funding for innovation

Across France, Germany, Italy, and the United Kingdom, hospitals are funded through fixed tariffs based on diagnosis-related groups (DRGs), which provide a fixed fee for treatment of patients with certain diagnoses and levels of complications. These tariffs are calculated retrospectively, based on actual cost data typically captured two or more years earlier. Hospitals in Spain operate with fixed annual budgets, which are negotiated with each regional health ministry, and are based on treatment volumes in previous years (8). Regardless of country, new therapies that enter the hospital setting at a premium price to that of existing treatments present a funding gap for hospitals that needs to be addressed in order to optimise adoption and market access of the new and more expensive therapy. Such additional funding mechanisms exist in all countries, however, to various extents.

In France, the Council of Hospitalisation (within the Ministry of Health, MoH) is in charge of maintaining the DRG framework for hospital funding and decides on which therapies to exclude, and fund separately, on top of the DRG tariffs; these DRG exclusion are called *liste en sus* or *hors T2A*. This mechanism of funding is restricted to high-cost therapies that are either 1) awarded ASMR I, II, or III *or* 2) awarded ASMR IV or V against a product with ASMR I-III (and is already on *liste en sus*). The decision for inclusion on the *liste en sus* is not very transparent, so lobbying and political relationships are instrumental parts of the process.

In Germany, two related funding mechanisms exist to address the funding gap where existing DRG tariffs are insufficient: 1) *NUB*, an intermediate funding mechanism for the first two years after launch (until DRGs tariffs are updated), and 2) *ZE*, a permanent DRG tariff top-up running beyond the two-year mark for therapies that are only used in a minority of centres and patients (and would therefore not be covered adequately even by the updated DRG tariffs). Hospitals apply individually for products to acquire *NUB* status of new therapies to the Institute for hospital remuneration (InEk), which administers the German DRG framework. InEk decides whether the new technology is eligible for the *NUB* status based on two main criteria: 1) The product must be provided in a very limited number of hospitals with a supra-regional catchment area and 2) the cost increase must exceed the existing tariff by >50% (28). Once the new treatment has been awarded the *NUB* status, hospitals enter into (annual) negotiations for actual payments with the sickness funds (health insurers), which are the ultimate decision-makers in providing additional funding. These negotiations are confidential and the agreed *NUB* tariffs can differ between hospitals for the same treatment, as negotiations are done individually for each hospital. However, when *NUB* is replaced by the permanent *ZE*, the same add-on tariff applies across all hospitals.

In Italy, each region is in charge of hospital funding and commonly apply their own, region-specific DRG tariffs (7). Funding on top of these tariffs may be granted by regions for high-cost drugs, by inclusion on the so-called «File F» list. Each region has its own File F list, and there is substantial variation between them in terms of which treatments are given this status. Local-level physicians initiate the File F application, which must be supported by the hospital pharmacy director; the application is then approved by the regional director of pharmacy services. Manufacturers play no direct role in this process and can mainly only supply data to ensure that the application is as robust as possible.

In Spain, annual budgets (based on past treatment volumes) are the norm for hospital funding, and it is the autonomous regions that allocate these budgets (8); this often translates into considerable discrepancies in hospital funding across Spain. Funding beyond the annual budget, for example, supplementary payments for premium-priced innovative therapies is provided only in exceptional cases (e.g., cases with high publicity or political pressure). Hospitals can apply to the regional authorities for additional funds, but there are no defined criteria for decision-making.

In England, the hospital remuneration system is called payment by results (PbR), and the tariffs are structured similarly to the DRGs, although they are called Healthcare Resource Groups (HRGs), as opposed to DRGs. Licensed ATMPs are commissioned through specialised services which are excluded from the standard HRG tariffs, and funded separately. These exclusions apply mainly to specialist therapies such as ATMPs, which are used only in a relatively small number of centres (rather than provided evenly across all trusts), targeting comparatively small number of patients (29).

### Impact of target population size

The size of the target population impacts P&R&MA in all the Big5EU countries, however, in slightly different ways.

In France and Germany, patient population size mainly affects P&R&MA indirectly, in that patient numbers drive annual sales revenues, which impact the price negotiations as well as the assessment rigour applied to therapies at launch. In France, population sizes drive the conditions of the price/volume agreements with the pricing authority (CEPS). Furthermore, for therapies that aspire to have ASMR I-III, only therapies that have an expected annual revenue of >€20 million are required to undergo the CE analysis by CEESP (30, 31). In Germany, therapies with orphan indications (and expected annual revenues <€50 million) are assumed inherently to have added benefit, are exempt from the early benefit assessment, and can therefore enter straight into price negotiations. Furthermore, therapies in any indication, with expected annual revenues <€1 million avoid both the early benefit assessment and national price negotiations, and can be priced freely (13).

In Italy and Spain, the P&R&MA impact of the target population size is less prescriptive; however, its impact on the healthcare budget is a major consideration in price negotiations at all levels. In Italy, all new medicinal products with an orphan or oncology indication are, at least initially, subject to so-called innovative P&R arrangements, negotiated with AIFA. In Spain, where additional funding for high-cost therapies is rare, acquiring such funding is thought to be more likely in smaller patient populations, where budget impact is more limited.

In England, high-cost therapies targeting populations smaller than 500 patients per annum in severely disabling conditions of high unmet need will likely be subject to the HSTE rather than the TA; however, the HSTEs only apply to therapies that are delivered on a chronic basis (5). Therefore, for ATMPS that are administered for a finite period in small patient populations, no NICE assessment exists that results in binding obligations for the NHS. Therapies without a binding NICE assessment are subject to assessment by the NHS specialised services. Furthermore, therapies that target less than 20 patients per year are funded through individual funding requests (rather than formal assessments) (5).

## Discussion

In the above paragraphs, we have detailed the ATMP-relevant differences in P&R&MA pathways and decision-making criteria across the Big5EU, and how this may differ for small patient populations. In the below paragraphs, we discuss some of the general challenges faced by manufacturers of ATMPs in realising the value potential of their therapies, and highlight relevant country-specific issues to inform risk-mitigating strategies.

### The role of clinical benefit analyses in P&R&MA

In all Big5EU countries, clinical benefit is measured in comparison to an appropriate comparator. This comparator may differ between countries (as clinical practice may be different in different geographies), which necessitates indirect comparisons (as incorporation of multiple comparator arms in ATMP pivotal trials is challenging due to recruitment hurdles arising from targeting niche populations). National decision-makers evaluate the degree of comparative benefit, and for undifferentiated products (i.e., comparable clinical benefit to the comparator), typically apply a domestic comparator price. In the following paragraphs, we will focus on the P&R&MA implications of differentiated products that provide an additional benefit compared with existing treatments.

France and Germany both have defined scales (ASMR scores and the level of added benefit respectively) that rate the incremental clinical benefit of new market entrants versus that of the comparator. In France, the ASMR score is of great strategic importance, not only because higher scores (ASMR I-III) typically allow for higher price points but also because hospital products with these scores are candidates for top-up funding (*liste en sus*) beyond the existing hospital tariffs (which removes the funding barrier for local-level uptake). In Germany, the rating framework for evaluating added benefit is relatively new (originating in 2011), and its implications in terms of price potential and funding are not clearly defined, which gives decision-makers flexibility to negotiate on a case-by-case basis. It should also be emphasised that when international price referencing is applied, Germany uses a lower-cost basket of countries (EU15) unlike France that references the more premium-priced BigEU4.

In Italy, AIFA decides whether the new treatment is considered ‘innovative’, which has important market access implications as all regions have to make innovative products available. AIFA introduced the *innovation algorithm* in 2007, as a framework to describe the factors that determine what product characteristics make a therapy innovative. While the original algorithm had three outcomes in terms of levels of therapeutic innovation (‘modest’, ‘moderate’, and ‘important’), this has since changed. However, the criteria currently used by AIFA are not available in the public domain at the time of writing. The most recently published list of innovative therapies (of 24 May 2015) (32) suggests that there are at least three levels: ‘Yes (innovative)’, ‘Important’, and ‘Potential’. However, how these different categories affect pricing is left to the discretion of the national and regional payers.

In Spain, there are no rating scales defined as part of the national product assessments. However in 14 of the 17 regions, a decision algorithm exists, which rates the new treatment's therapeutic improvement on a scale of 0–4, depending on the degree of improvement observed, and the robustness of the data. Similarly as in Italy, the application of this algorithm, and how it translates into price is at the discretion of regional payers, who retain the flexibility to negotiate individually.

In England, the degree of improvement in clinical benefit also plays a central role in the value potential of new treatments, and it is commonly expressed in terms of QALYs gained.

### The role of budget impact analyses in P&R&MA

Treatment cost is a key consideration for market access decision-makers, regardless of geography. Willingness to pay is typically higher in smaller patient populations, especially in populations with high disease burden. This is exemplified by the reimbursement restrictions imposed on proprietary biologics in autoimmune disease such as rheumatoid arthritis across the major European healthcare systems; such restrictions have narrowed the use to refractory patients failing lower-cost therapeutic options (33).

Although affordability is key to the sustainability of healthcare systems, budget impact analysis can pose a challenge to rewarding and incentivising innovation due to two common limitations: 1) the focus of the analysis is typically the healthcare budget in isolation, meaning savings in the social care budget (e.g., rehabilitation or long-term social care) are inadequately captured when benefits are assessed and 2) the time horizon of the analysis tends to be 1 or 2 years, meaning long-term benefits are also inadequately captured.

These challenges present across all Big5EU countries to different extents, however, are most pronounced in Italy and Spain, where public healthcare budgets are particularly constrained.^4^ In these countries, the decision to adopt a new treatment is greatly influenced by the short-term budget impact. Budget impact considerations also vary within individual countries, with local-level adoption decisions being more sensitive to short-term budgetary implications.

### The role of CE analyses in P&R&MA

CE (or cost utility) analysis is increasingly gaining traction as a means to evaluate health interventions across Europe. Still, in the Big5EU, it is only the United Kingdom that links the cost per QALY specifically and methodologically to P&R&MA. CE analysis is now also a mandatory part of product evaluation in France and Spain, although it performs a more well-defined function in the former than in the latter. In France, CE analysis is used as a lever to negotiate prices for innovative therapies with a substantial clinical benefit (ASMR I-III), using the EU4 price corridor as a benchmark. In Spain, CE is mandatory for the national submission, and increasingly also considered by the regional authorities; however, the impact on P&R&MA is not well defined.

In Italy, CE analyses play no part in the national evaluation, and only a handful of regions (e.g., Veneto, Emilia Romagna, and Tuscany) use this in decision-making (7). The application of this methodology is still novel in the Mediterranean Big5EU countries, and it plays mainly a supplementary role to that of the conventional evaluation of comparative efficacy and budget impact, meaning that treatments that are cost-effective may be restricted or denied reimbursement due to budget concerns.

The United Kingdom, comprising its four devolved regions, is the only country that has explicit threshold values for CE of new treatments, ranging from £20,000 to £30,000 per QALY for indications with 500 or more eligible patients, and up to £50,000 for end-of-life indications. In very rare conditions, these thresholds are less relevant, as is illustrated by the example of Cerezyme in Gaucher's disease (in a patient population of approximately 270), where the treatment was found to have a cost per QALY (ICER) of £391,244 (34). While CE is a key component in P&R&MA, it is not a lever used in isolation, and must be considered in conjunction with practical and ethical considerations, which can alter the outcome from a P&R&MA perspective. A therapy that is not cost-effective from a NICE, SMC, or AWMSG perspective is not necessarily excluded from commissioning; however, the commercial risks of not being adopted by the NHS are far higher in this scenario. Therefore, manufacturers should strive to present data that support a cost-effective price in order to maximise the commercial opportunity and minimise the risk of failure.

In Germany, CE analysis is largely unused, meaning comparative efficacy and budget impact remain the key decision-making criteria for P&R&MA. A cost-benefit analysis is possible as a last resort in cases where both the price negotiations and arbitration have failed. However, this analysis differs substantially from CE in terms of methodology. One key issue with the CBA methodology is that it yields different cost-benefit frontiers (i.e., willingness to pay) in different therapy areas, because the cost-benefit frontier is derived from the differences in net costs and benefits of treatments in use in the specific therapy area. Although this methodology can be useful to elicit willingness to pay in a specific therapy area based on current practice, it falls short of providing a coherent framework to apply across a healthcare system in order to ensure equitable access to care.

Although CBAs are a theoretical possibility, they have not been applied in practice since the introduction in 2011. Presently, it therefore remains a theoretical exercise more than a decision-making tool to be reckoned with. The reasons for this are unclear; however, the lack of examples from practice suggest that both decision-makers and manufacturers favour the conventional assessment methodology.

### The role of outcomes modelling in P&R&MA

Generating adequate data for P&R&MA during clinical development is one of the main challenges for manufacturers, and is an even greater challenge in the case of many ATMPs. The high cost of goods for ATMPs increase the financial burden of conducting trials, as well as necessitating a higher price point in order to be commercially viable. Manufacturers therefore often target indications with a high unmet need and a relatively small number of patients; this is because the potential clinical benefit is greater, and a smaller trial size and higher costs of care may be considered more acceptable by payers.

Conducting trials in less prevalent disease areas with high unmet need presents substantial challenges in terms of evidence generation. Low patient numbers mean that recruitment of enough subjects to power the trial sufficiently is time consuming, which erodes the patent protection time. Also, in therapy areas with a high unmet need (e.g., lacking effective therapy options and high morbidity and/or mortality), it may not be ethically justifiable to randomise patients to receive placebo as a control, when an efficacious alternative exists. Such situations necessitate single-arm trials, which are considered lower grade evidence from a payer perspective, as it can only be compared indirectly with the current SOC, which makes P&R&MA negotiations more challenging. Furthermore, long-term benefits cannot be captured within the timeframe of a clinical trial, meaning that a substantial part of the value proposition remains undocumented in the case of treatments with long-term benefits (e.g., curative treatments).

Outcomes modelling aims to bridge the gap that presents itself in the absence of perfect information from trial or real-life data. For many ATMPs, indirect comparisons and extrapolations are particularly relevant, in the light of the challenges described above.

Indirect comparison is a methodology used to compare data from different sources where appropriate direct comparisons are not available. This methodology is particularly relevant for ATMPs in two scenarios: 1) where the comparator in the pivotal trial does not reflect the SOC in the country in question, or 2) where ethical considerations dictate a single-arm study. Indirect comparisons provide a way to utilise data from other studies or observational sources (meta analyses, registries, etc.) to estimate the comparative effectiveness of the new treatment.

The nature of this methodology means that there will be discrepancies in the patient populations in which effectiveness is measured, either because of differences in inclusion criteria of the studies or because observational studies are conducted in an uncontrolled environment as opposed to a controlled trial. This creates an imbalance in the data, which reduces the robustness of the analysis used to estimate the relative treatment effects. Statistical regression methods can help control these imbalances as well as adjust survival estimates in the presence of treatment switching, and provide more robust estimates for decision-makers to use in their evaluations.

In the United Kingdom, NICE, SMC, and AWMSG accept indirect comparisons and statistical regression modelling as a basis to make recommendations in situations where there is a justified absence of RCT data. However, manufacturers should be discouraged from modelling outcomes obtained in a certain therapeutic position to forecast effectiveness in a different one (e.g., from first to second or third line), as proportional hazards across different lines of therapy cannot be assumed.

Extrapolation of longer-term effectiveness from shorter-term clinical trial data is commonly used to estimate the treatment effectiveness beyond the clinical trial period. The methods used to do this include the development of multiple parametric and semi-parametric models, which are subsequently validated on the grounds of statistical considerations and clinical expert opinion on biological plausibility. These methods can form the basis for health economic models, which can estimate the lifetime costs and health outcomes of the therapy in question. Across the Big5EU, only NICE in England provides a clear HTA guidance on how long-term claims can be substantiated through extrapolation; however, the acceptability of such methods by other European HTA bodies is not currently established (35).

### Dealing with uncertainty in P&R&MA

Both indirect comparisons and extrapolations are associated with uncertainty regarding the modelled results, depending on factors such as the statistical significance in the original RCT data and the relative length of the extrapolation in comparison to the RCT follow-up period. Deterministic, probabilistic, and structural sensitivity analysis provide useful tools to assess the impact of these uncertainties on the value of the future claims.

RSAs between the manufacturers and payers can help mitigate this uncertainty and can in combination with real-world evidence generation provide a vehicle for rewarding the full benefits of ATMPs while limiting the risk and financial exposure for payers. However, such schemes necessitate regular patient follow-up and are often associated with significant clinical and administrative burden, which has limited their implementation and favoured confidential discounts instead. Therefore, manufacturers should carefully consider whether they wish to avoid agreeing to an upfront discount by taking a share of the administrative burden of the RSA in return for the opportunity to capitalise on the full benefits of the licensed ATMP.

Outcomes-based RSAs can take a number of different forms, but typical components often include:

1. Price increases or decreases based on real-world evidence
  - For example, rebates or paybacks apply if the observed treatment effect is lower than in the pivotal data
2. Payment structure
  - Upfront
  - Annuity based
    - As savings or benefits materialise
    - In agreed instalments (independent of benefit)

#### Cohort versus individual patient level

Italy has historically been a keen adopter of RSAs compared with the other Big5EU countries, and payment for responders only is commonly applied in oncology and rare diseases. In these cases, funding and use is typically restricted to certain centres, and patient outcomes must be recorded in product-specific AIFA registries, at the expense of the manufacturer. Establishing registries for a new product can be a hurdle and can delay actual adoption, and also come at a fee of €30,000, levied by AIFA, per registry per year. Such agreements will be likely to apply to ATMPs as well.

In terms of examples of RSAs for ATMPs specifically, the limited number of licensed products on the market means that there are very few to choose from. The only example found in our research across the Big5EU was that of ChondroCelect in Spain, which was a payment-for-performance scheme, on an individual patient basis, where Spanish payers would get a 100% refund if the treatment failed after year one, 75% for failure at year two, or a 50% refund for failure at year three (18).

RSAs are also applied in the United Kingdom through the PASs (although these are rare and mainly in oncology), and unlike discounts (which are kept confidential), the details of outcomes-based agreements are disclosed. For example, Velcade in progressive multiple myeloma: manufacturer rebates the full cost of Velcade for people who, after a maximum of four cycles of treatment, have less than a partial response (defined as reduction of serum M protein by ≤50%) (36).

In France, the use of price-volume agreements is far more prolific than RSAs; however, this may change as new treatments that challenge the standard P&R framework are launched.

RSAa are not possible at the national level in Germany, and although there are examples of regional agreements (37, 38), simple discounts are typically preferred by the individual sickness funds.

### The role of healthcare delivery configuration

Another potential barrier for ATMP adoption is that some of these treatments change the patient pathway, and the way treatments are delivered; national health service organisations’ ‘readiness’ to adopt novel technologies, or ability to adapt existing infrastructure and care delivery, can pose hurdles to adoption (39).

For both autologous and allogeneic treatments, the main issue from a service configuration perspective is whether the necessary facilities are in place for product use, or whether complex storage and pre-administration preparations are needed, for example, thawing as well as requirement for complex interventional procedures and capital infrastructure.

Adopting autologous therapies in particular will in many cases mean that patients require an additional outpatient appointment (as patients would need to undergo separate appointments for the cell harvest *and* the re-infusion as opposed to just one appointment for administration for most conventional therapies). This not only adds costs, which affects the budget impact and CE analyses, but also reduces the flexibility of hospitals in scheduling consultations, as the limited shelf life of cell products means patients need to be seen again within a limited timeframe. This puts pressure on the healthcare service configuration to adapt to the new patient pathway, which is a challenge both from an organisational and financial perspective. The highly specialised nature of some of the resources and capabilities required to handle cell therapy products will require workforce development, education, quality standards, and accreditation, and potential investments in devices or equipment to facilitate the procedures. Furthermore, healthcare providers need to plan to ensure that there is adequate capacity in clinical services such as apheresis units, inpatient beds, and intensive care units to facilitate appropriate supportive care.

## Conclusions

The high cost of ATMPs, coupled with the uncertainty at launch around their long-term claims, presents challenges for their adoption at a commercially viable reimbursed price. Early assessment and shaping of the pricing and reimbursement potential is needed from the pre-clinical stage. It is critical to understand the disease burden and room for innovation, and how these vary across the different therapeutic positions in the treatment algorithm and subpopulations, to inform the development of positioning strategies. Furthermore, identification of the key health economic drivers and their inclusion in the target product profile can help maximise the value proposition and guide clinical development; in addition, identification of the minimum efficacy thresholds required to support a commercially viable reimbursed price can be used to inform *go/no-go* decisions as clinical evidence is being generated. Overall, in order to maximise the likelihood of achieving reimbursement at a commercially viable price level, it is important to ensure that the incremental benefit of the novel ATMP is proportionate to its incremental cost above current therapeutic approaches by accounting for differences in individual country assessment frameworks and value drivers. In this respect, populations of high disease burden and unmet need may be best targeted, as the potential for improvement in patient benefit is greater, as well as the potential for capitalising on healthcare cost offsets. Targeting small populations can also help reduce both payers’ budget impact concerns and the risk of reimbursement restrictions being imposed, especially at a local level where the ability to absorb additional costs can impact uptake. Given that a key feature of ATMPs’ value proposition is claims on long-term benefits from a single (or limited number of) treatment, the challenges with substantiating such claims at launch is significant; outcomes-based risk-sharing schemes coupled with real-world evidence generation provide opportunities to manage uncertainty and reward for the full benefits of the ATMPs. Such schemes in combination with annuity-based payments can also help minimise payers’ budget impact concerns. However, manufacturers need to play a central role in facilitating the implementation and alleviate the administrative burden to healthcare systems in order to encourage their usage.

## Footnotes

1 NICE guidance on service developments and therapies considered to be suitable for reimbursement within the NHS extend to Northern Ireland, albeit being subject to local review and funding.

2 The early benefit assessment is a central part of the AMNOG P&R reform, which was introduced in 2010.

3 All interventions with an ICER below £20,000 are recommended for NHS adoption; as the ICER of an intervention increases towards £30,000, the evaluation committee will consider in more detail the degree of certainty around the ICER, the adequacy of the quality of life benefit, the innovative nature of the technology, whether the technology is considered to be a ‘life-extending treatment at the end of life’, and aspects that relate to non-health objectives of the NHS.

4 In Spain, drug spending has been reduced by 30% in the past three years, which illustrates the impact of cost minimisation in this country.
